# Nicotine Enhances Amplitude and Consistency of Timing of Responses to Acoustic Trains in A1

**DOI:** 10.3389/fncir.2021.597401

**Published:** 2021-02-18

**Authors:** Irakli Intskirveli, Raju Metherate

**Affiliations:** Department of Neurobiology and Behavior, Center for Hearing Research, University of California, Irvine, Irvine, CA, United States

**Keywords:** nicotine, auditory cortex, hearing, mouse, frequency following, current source density

## Abstract

Systemic nicotine enhances neural processing in primary auditory cortex (A1) as determined using tone-evoked, current-source density (CSD) measurements. For example, nicotine enhances the characteristic frequency (CF)-evoked current sink in layer 4 of A1, increasing amplitude and decreasing latency. However, since presenting auditory stimuli within a stream of stimuli increases the complexity of response dynamics, we sought to determine the effects of nicotine on CSD responses to trains of CF stimuli (one-second trains at 2–40 Hz; each train repeated 25 times). CSD recordings were obtained using a 16-channel multiprobe inserted in A1 of urethane/xylazine-anesthetized mice, and analysis focused on two current sinks in the middle (layer 4) and deep (layers 5/6) layers. CF trains produced adaptation of the layer 4 response that was weak at 2 Hz, stronger at 5–10 Hz and complete at 20–40 Hz. In contrast, the layer 5/6 current sink exhibited less adaptation at 2–10 Hz, and simultaneously recorded auditory brainstem responses (ABRs) showed no adaptation even at 40 Hz. Systemic nicotine (2.1 mg/kg) enhanced layer 4 responses throughout the one-second stimulus train at rates ≤10 Hz. Nicotine enhanced both response amplitude within each train and the consistency of response timing across 25 trials. Nicotine did not alter the degree of adaptation over one-second trials, but its effect to increase amplitudes revealed a novel, slower form of adaptation that developed over multiple trials. Nicotine did not affect responses that were fully adapted (20–40 Hz trains), nor did nicotine affect any aspect of the layer 5/6 current sink or ABRs. The overall effect of nicotine in layer 4 was to enhance all responses within each train, to emphasize earlier trials across multiple trials, and to improve the consistency of timing across all trials. These effects may improve processing of complex acoustic streams, including speech, that contain information in the 2–10 Hz range.

## Introduction

Activation of nicotinic acetylcholine receptors (nAChRs) increases neural excitability due to the influx of cations through the receptor ion channel (Dani and Bertrand, 2007; Albuquerque et al., 2009). However, the effect of nicotine on neural processing cannot be inferred from this cellular action alone since nAChRs are found on both excitatory and inhibitory neurons and in different neural compartments, regulating, for example, presynaptic release of neurotransmitter, postsynaptic depolarization, and action potential propagation along axons (Dani and Bertrand, 2007; Albuquerque et al., 2009; Poorthuis et al., 2013). Since sensory-evoked current-source density (CSD) profiles reflect integrated synaptic activity within neural circuits, they provide a circuit-level measure that can be used to evaluate nicotinic regulation of neural processing (Muller-Preuss and Mitzdorf, 1984; Metherate et al., 2012). In primary auditory cortex (A1) of rodents, for example, nicotine enhances the characteristic frequency (CF)-evoked thalamocortical response (layer 4 current sink), increasing peak amplitude and decreasing both onset and peak latencies (Intskirveli and Metherate, 2012; Askew et al., 2017). This effect is likely due to multiple nAChR-mediated cellular actions in A1, including increased excitability of thalamocortical axons, excitation of a subset of inhibitory interneurons, and depolarization of pyramidal neurons due to disinhibition (i.e., excitation of interneurons that innervate other interneurons) (Kawai et al., 2007; Intskirveli and Metherate, 2012; Askew et al., 2019).

However, acoustic stimuli rarely occur in isolation and can trigger complex response dynamics when presented within a stream of auditory stimuli (Todorovic et al., 2011; Phillips et al., 2017). A simple example is the response adaptation that occurs when a stimulus is presented repetitively; i.e., evoked responses become progressively weaker during a train of CF stimuli, with the degree of adaptation increasing with repetition rate. Response adaptation is weak in the lower auditory pathway and increasingly prominent in the auditory forebrain, especially cortex. In A1, CF-evoked responses begin to adapt at very low repetition rates, e.g., 1–2 Hz, and adapt fully at rates of 15–20 Hz (Creutzfeldt et al., 1980; Wang et al., 2008; Yao et al., 2015).

Since repetitive stimulation produces strong adaptation in A1 and systemic nicotine enhances cortical responses, here we examined the effects of nicotine on response adaptation during CF stimulus trains (one-second trials of 2–40 Hz stimuli; trials repeated 25 times). For the CF-evoked current sink in layer 4, nicotine enhanced responses throughout the stimulus train at rates ≤10 Hz. Nicotine increased response amplitude, and notably, also enhanced the consistency of response timing. While nicotine did not affect the *degree* of adaptation over one-second trials, the drug revealed a novel, slower adaptation that emerged over multiple trials after initial response enhancement. The overall effect of nicotine was to enhance all responses within each train, to emphasize earlier trials across multiple trials, and to improve the consistency of timing across trials.

## Materials and Methods

### Animal Preparation

Adult (60–80 days old) male FVB mice were used for all procedures in accordance with the National Institutes of Health Guide for the Care and Use of Laboratory Animals and as approved by the University of California, Irvine Institutional Animal Care and Use Committee (IACUC). Mice were anesthetized with urethane (Sigma; 0.7 g/kg i.p.) and xylazine (Phoenix Pharmaceutics; 13 mg/kg i.p.) in saline, placed in a sound-attenuating chamber (model AC-3, IAC, Bronx, NY, United States) and maintained at 36–37°C. Anesthesia was supplemented as necessary (0.13 g/kg urethane, 1.3 mg/kg xylazine i.p.) via a catheter to avoid movement of the mice. The head was secured in a stereotaxic frame (model 923, Kopf Instruments, Tujunga, CA, United States). After a midline incision, the skull was cleared and secured to a custom-made head holder. A craniotomy was performed over the right auditory cortex and the exposed brain was kept moist with warm saline. A burr hole was made over vertex and a dental screw with connector inserted for recording the auditory brainstem response (ABR).

### Electrophysiology

For mapping A1, stimulus-evoked local field potentials (LFPs) were recorded with a glass micropipette filled with 1 M NaCl (∼1 MΩ at 1 kHz). ABR and LFP recordings were filtered and amplified (1–1000 Hz, AI-401, CyberAmp 380; Axon Instruments), digitized, and stored on a computer (AxoGraph software). LFPs for CSD profiles were recorded using a 16-channel silicon multiprobe (∼2–3 MΩ at 1 kHz for each 177-μm^2^ recording site, 100-μm separation between recording sites; NeuroNexus Technologies), filtered and amplified (1 Hz to 10 kHz, AI-405, CyberAmp 380), digitized and stored on a computer.

### Acoustic Stimulation

Acoustic stimuli were digitally synthesized and controlled with custom software and delivered through an open-field speaker (FF-1 with SA1 amplifier and RP2.1 Real Time-Processor; Tucker-Davis Technologies) positioned ∼3 cm in front of the left ear. For calibration [sound pressure level (SPL), in dB re: 20 μPa] a microphone (model 4939 and Nexus amplifier; Bruel and Kjaer) was positioned in place of the animal at the tip of the left earbar. For mapping A1, tones were 100 ms in duration with 5-ms linear rise and fall ramps (range 5–40 kHz and 0–70 dB SPL). For determining ABR threshold, white-noise stimuli (10 ms duration, 3 ms rise/fall ramps) were delivered at 2/s for 100 repetitions and repeated at 0–70 dB SPL. For multiprobe recordings, 10 ms tones (3 ms rise/fall ramps) were delivered at 2, 5, 10, 20, and 40 Hz for 1 s trials in sets of 25 trials at 30 dB above threshold.

### Determining the A1 Recording Site

To find a recording site in A1 we used our method previously described (Intskirveli and Metherate, 2012). Briefly, we recorded tone-evoked responses from multiple sites ∼250 μm apart along the anterior-posterior axis in auditory cortex at a depth of ∼400 μm (approximately layer 4). Based on responses to a standard set of tones (5–40 kHz in 2.5-kHz steps, 0–70 dB SPL in 5-dB steps), we determined CF (frequency with the lowest threshold) for each recording site. After constructing a CF map and confirming the tonotopy expected for A1, including a reversal of tonotopy at the border with the anterior auditory field (Stiebler et al., 1997), we chose a region within A1 and mapped along the dorsoventral axis to identify a recording site (CF 10–20 kHz) with a short-latency, large-amplitude response in layer 4 for all subsequent procedures. At this site we inserted a 16-channel multiprobe perpendicular to the pia surface to record LFPs throughout the cortical depth and re-determined CF (1-kHz steps) and threshold (5-dB steps) based on LFPs at a depth of 300–400 μm. Threshold responses exceeded three standard deviations of the mean baseline determined over 100 ms preceding the tone.

### Drug Application

(−)-Nicotine hydrogen tartrate (Sigma) was dissolved in saline, adjusted to pH 7.0 and delivered subcutaneously (2.1 mg/kg, free base). This dose is reliably suprathreshold for nicotine effects in mouse A1 (Intskirveli and Metherate, 2012).

### Data Analysis

For each one-second trial, tone-evoked LFP responses were baselined using the 10 ms period before the first stimulus, and one-dimensional CSD profiles constructed off-line using custom Matlab script. CSD profiles are the second spatial derivative of the LFP laminar profile (Muller-Preuss and Mitzdorf, 1984); conventionally, a current sink implies the location, timing, and magnitude of underlying synaptic excitation. In each CSD profile we identified two prominent current sinks based on onset latency and depth. First, the current sink in the middle layers (typically 200–400 μm depth) with shortest onset latency was designated the “layer 4” current sink. A second, deeper current sink, typically 300 μm below the layer 4 sink, was designated the “layer 5/6” current sink. Current-sink peak amplitudes and latencies (for the max peak within 100 ms from stimulus onset) were measured in each condition. Coefficient of variance was calculated to show changes in latency variability after nicotine injection. Adaptation ratio during a train stimulus was calculated as the peak amplitude of the mean adapted response divided by the first response (“mean adapted response” is 2nd response for 2 Hz, mean 3rd–5th response for 5 Hz and mean 3rd–10th response for 10 Hz). Statistical comparisons were performed with GraphPad Prism. Related means (for pre-drug, saline, and nicotine responses) were compared using repeated measures (RM-) ANOVA and Tukey’s *post hoc* test for multiple comparisons. Since for each mouse all data were obtained at a single recording site, for group statistics “n” refers to the number of animals.

## Results

After mapping with a microelectrode to determine the location of A1 in each animal, we selected a single recording site from among those exhibiting robust CF-evoked LFPs in the middle layers. The selected recording sites exhibited CFs of 10–20 kHz. At the selected site we inserted a 16-channel linear multiprobe to record LFPs and derive CSD profiles. We analyzed two prominent CF-evoked current sinks: the shortest-latency middle-layer current sink (“layer 4”) and the infragranular sink in layer 5/6. For stimulus trains, CF tones (10 ms duration, 30 dB above threshold) were presented at each rate (2, 5, 10, 20, and 40 Hz) for one second, repeated 25 times (2.5 s between trial onsets, total duration of each set ∼1 min). Stimulus sets for each rate (2–40 Hz) were presented in random order and the entire series (five rates) repeated so that results for each rate were averaged from two stimulus sets per condition. Stimulus sets were presented under three conditions: before any manipulation (Pre), after systemic saline (Saline) and after systemic nicotine (Nicotine; 2.1 mg/kg, s.c.). The total duration of acoustic stimulation in each condition was ∼12–15 min, which is less than the typical duration (∼30 min) of nicotine effects from a single injection (Intskirveli and Metherate, 2012).

**FIGURE 1:**
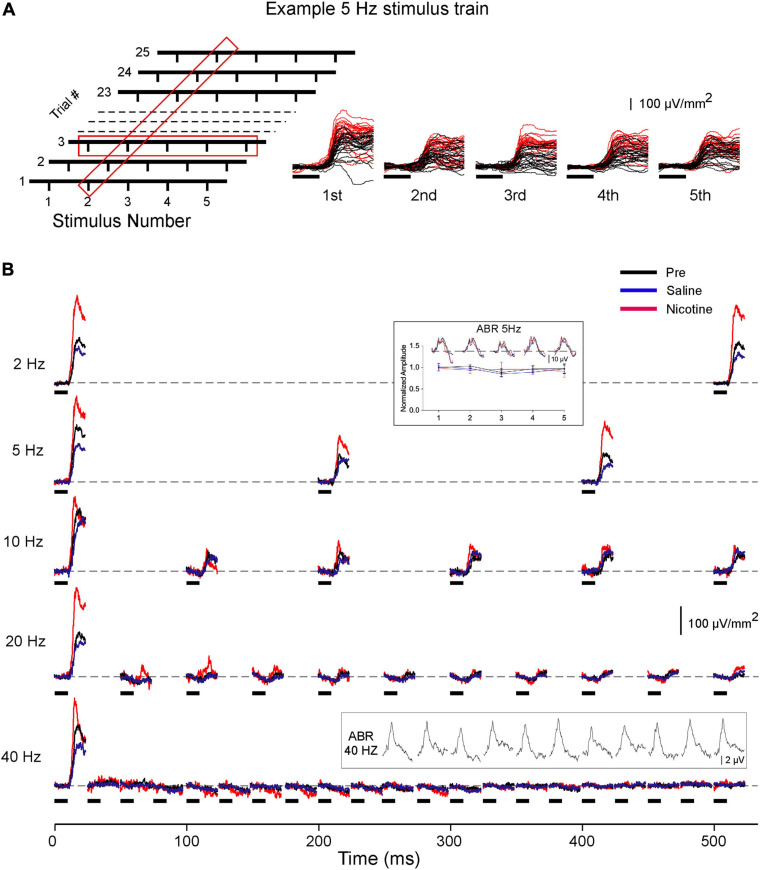
Effects of systemic nicotine on layer 4 current sink evoked by CF stimulus trains. **(A)** Schematic of 5 Hz stimulus set (left): 25 trials, each with five CF stimuli presented over one second; 1.5 s inter-trial interval. Red boxes represent data averaged across 25 trials, as in panel B and Figures 2, 4, or averaged within each trial, as in Figure 3. Traces (right) show example 5 Hz responses in pre-drug condition (black traces) and after systemic nicotine (2.1 mg/kg; red traces). In this and the following figures, horizontal marks indicate 10 ms tone presentation. **(B)** Representative layer 4 current sinks evoked by CF stimuli presented at different rates (2–40 Hz); responses shown are for the first half of each 1-s trial and traces are average from two sets of 25 trials. Inset above 5 Hz response shows simultaneously recorded ABR (example traces) and group data for ABR peak amplitude in each condition. Inset above 40 Hz response shows separately recorded ABR (10 ms white noise stimulus).

Figure 1A depicts one stimulus set for a 5-Hz CF train (left) alongside a representative set of responses (right) showing the layer 4 current sink evoked in the Pre (black traces) and Nicotine (red traces) conditions. Averaged responses for this animal are in Figure 1B and exhibit typical response adaptation in the pre-drug condition (each trace is average of two sets of 25 trials): adaptation was minimal at 2 Hz but increased with stimulus rate until complete adaptation occurred at 20 and 40 Hz. Inset traces above 5 and 40-Hz responses show ABR recordings that exhibited no adaptation even at 40 Hz. Since, in most animals, stimulus rates of 20–40 Hz produced complete adaptation of cortical responses, only data for rates up to and including 10 Hz were analyzed for effects of nicotine. Data were obtained from 10 mice, but three early experiments did not include 2 Hz stimulation. In four animals, 10 Hz produced complete adaptation and the results were not analyzed further. One experiment had a faulty ABR electrode and was excluded from ABR analysis.

### Systemic Nicotine Enhances Layer 4 Response to CF Stimulus Trains

**FIGURE 2:**
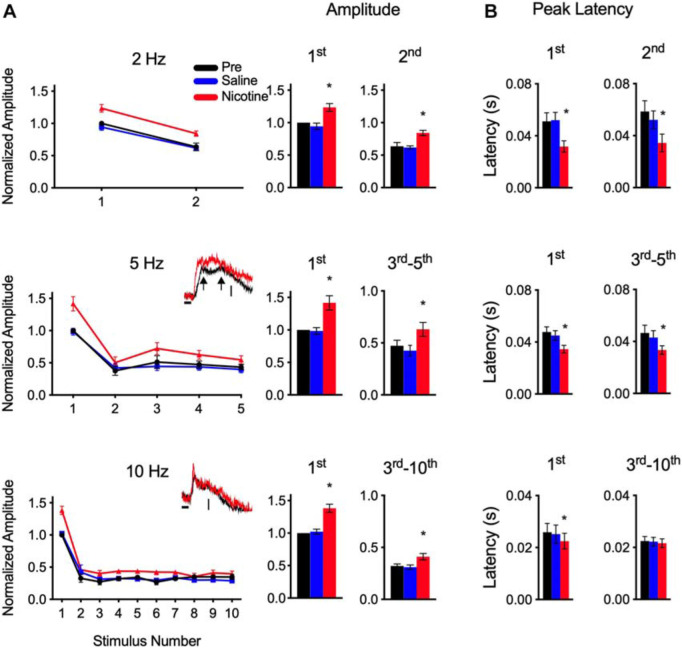
Nicotine increased peak amplitude and decreased peak latency for responses to CF stimulus trains. **(A)** Group data show peak amplitude (normalized to value of first pre-drug response) for trains at 2, 5, and 10 Hz. Insets for 5 and 10 Hz show example traces for adapted responses, vertical scales represent 25 mV/mm^2^. Histograms show amplitudes separately for first response and adapted responses combined. In this and following figures, *indicates *p* < 0.05. **(B)** Group data show peak latency separately for first response and adapted responses.

Consistent with previous studies (see section “Introduction”), systemic nicotine enhanced the layer 4 response to the first stimulus in each train. The example in Figure 1B and group data in Figure 2 show that nicotine increased the peak amplitude of each first response compared to pre-drug and saline responses and reduced its peak latency. For subsequent responses within each train, nicotine similarly enhanced response amplitude despite adaptation that increased with stimulus rate (Figures 2A,B; inset traces are examples of adapted responses at 5 and 10 Hz). When normalized to pre-drug amplitude, nicotine’s enhancement of adapted responses was similar to its effect on the first response (Figure 2; first response to 2–10 Hz trains enhanced 32–44%, adapted responses enhanced 33–60%). Fully adapted responses at the highest rates (20–40 Hz) were not enhanced by nicotine (Figure 1B). Systemic saline had no effect on any measure (Figures 1, 2, blue traces, graphs, and histograms).

Graphs in Figure 2A (left), suggest enhancement of peak amplitude for nicotine compared to pre-drug and saline responses. For statistical analysis (Figure 2A, right), data are grouped separately for the first response at each rate and for subsequent adapted responses (for this and the following analyses, data for 5 and 10 Hz exclude 2nd response due to variable reduction before a plateau level of adaptation); asterisks indicate significant enhancement compared to pre-drug and saline values (RM-ANOVA with Tukey *post hoc* tests; 2 Hz: 1st response *p* = 0.0004, 2nd response *p* = 0.0020, *n* = 7; 5 Hz: 1st response *p* = 0.0009, 3rd–5th response *p* = 0.0035, *n* = 10; 10 Hz: 1st response *p* = 0.0005, 3rd–10th response *p* = 0.0023, *n* = 6). *Post hoc* tests confirmed no effect of saline on any measure of amplitude or latency (p’s ≫ 0.05).

Despite enhanced amplitudes, nicotine did not alter the *degree* of adaptation as estimated by the ratio of adapted responses to the first response. This adaptation ratio for pre-drug responses averaged 0.67 ± 0.04 for 2 Hz, 0.43 ± 0.04 for 5 Hz and 0.3 ± 0.02 for 10 Hz and did not change with saline or nicotine (RM-ANOVA, p’s ≫ 0.05). Thus, nicotine enhanced response amplitudes, but did not affect adaptation, within each stimulus train.

Simultaneous ABR recordings showed no effect of nicotine or saline on brainstem responses (Figure 1B, inset data at 5 Hz; peak amplitude averaged for 1st–5th response; RM-ANOVA, *p* = 0.331, *n* = 9), indicating that the locus of nicotine’s effect is more central.

As in prior studies, nicotine reduced the peak latency of the first response at all rates (Figure 2B; RM-ANOVA, 2 Hz: *p* = 0.0065, *n* = 7; 5 Hz: *p* = 0.0051, *n* = 10; 10 Hz: *p* = 0.0020, *n* = 6). Note, as shown in Figure 2A, inset traces, that adapted responses to 5 Hz trains (and 2 Hz, not shown) had longer-latency peaks than did 10 Hz responses, and at times exhibited two peaks, at short and long latencies (arrows in inset traces, Figure 2A). In contrast, 10 Hz responses exhibited only short-latency peaks (inset traces, Figure 2A). Since short and long latency peaks were not always evident, only a single value (max peak) was used for analysis. For 2 Hz and 5 Hz adapted responses, max peaks were altered by nicotine, exhibiting larger amplitudes (above, Figure 2A) and shorter latencies (Figure 2B; RM-ANOVA; 2 Hz: 2nd response *p* = 0.0120, *n* = 7; 5 Hz: 3rd–5th response *p* = 0.0065, *n* = 10). However, for the 10 Hz responses, nicotine had no effect on peak latencies of adapted responses (3rd–10th response *p* = 0.447, *n* = 6). The 10 Hz data likely reflect adaptation of longer-latency response components that did not recover between stimulus trials, leaving only a shorter-latency peak (latency ∼20 ms) that was not affected by nicotine.

Thus, nicotine enhanced partially adapted responses throughout one-second stimulus trains at 2–10 Hz by increasing peak amplitude, and for 2–5 Hz trains also reduced peak latency. However, nicotine did not change the *degree* of adaptation and did not affect fully adapted responses at 20–40 Hz.

### Nicotine Improves Timing Consistency of Layer 4 Response to Repeated Trials

**FIGURE 3:**
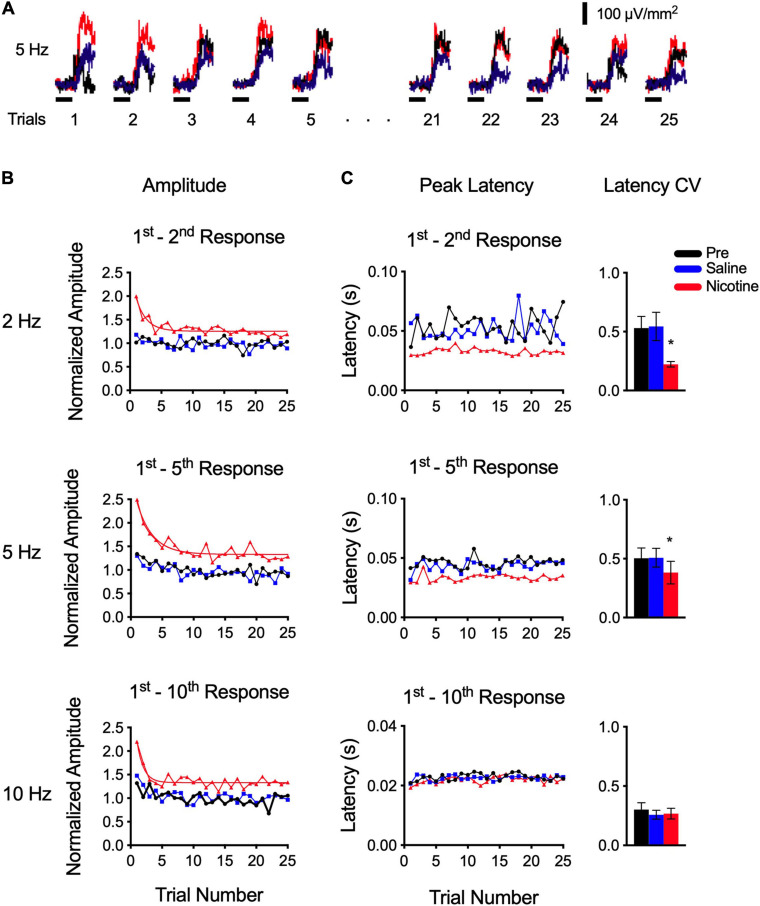
Nicotine enhanced responses across 25 trials—preferentially for initial trials—and improved timing consistency. **(A)** Example response to 5 Hz stimulus set. Each trace is average of all five responses within a trial (see horizontal red box in Figure 1A); figure depicts responses across 25 trials. **(B)** Group data show that nicotine enhanced response amplitudes for 2–10 Hz trains (as expected, cf. Figure 2A), but enhancement was greater early in the stimulus set, whereas pre-drug and saline controls show little change over 25 trials (group data are average of two stimulus sets in each of 6–10 animals; for clarity, error bars are not shown). **(C)** Nicotine reduced peak latency (as expected, cf. Figure 2B) and reduced latency variability across 25 trials (left), as reflected in coefficient of variation (right), for 2 and 5 Hz trains.

We next examined the degree to which nicotine altered response consistency from trial to trial (over 25 trials). As illustrated schematically in Figure 1A, this analysis involved averaging all responses *within* each trial (i.e., averaging 2, 5, or 10 responses per trial) and plotting the result for the 25 trials in each stimulus set. An example for a 5 Hz stimulus set is in Figure 3A and group data are in Figures 3B,C. For peak amplitude (Figure 3B), control responses (pre-drug and saline) exhibited little change over 25 trials, suggesting weak or no effects of stimulation that outlasted the 1.5 s interval between the end of one trial and the beginning of the next (pre-drug adaptation ratio for trials 20–25: 2 Hz: 0.95 ± 0.07; 5 Hz: 0.92 ± 0.07; 10 Hz: 1.13 ± 0.16). Nicotine, however, revealed an additional effect: while the drug increased response amplitude across all 25 trials, its effect was more prominent in early trials (Figure 3B). At each rate, nicotine enhanced response amplitudes for both the first six and last six trials in the stimulus set (RM-ANOVA; 2 Hz: first six trials *p* = 0.0005, last six trials *p* = 0.0203, *n* = 7; 5 Hz: first six trials *p* = 0.0048, last six trials *p* = 0.0028, *n* = 10; 10 Hz: first six trials *p* = 0.0119, last six trials *p* = 0.0154, *n* = 6). And, for 2 and 5 Hz stimuli, the increase in amplitude for the first six responses was greater than that for the last six responses (*t*-tests, 2 Hz: *p* = 0.0066; 5 Hz: *p* = 0.0062; 10 Hz: *p* = 0.983), indicating a more prominent effect of nicotine early in the stimulus set. Similarly, for 2 and 5 Hz stimuli the initial response in nicotine showed greater adaptation than in controls (RM-ANOVA, 2 Hz, *p* = 0.009; 5 Hz, *p* = 0.0139; 10 Hz, *p* = 0.925). Overall, nicotine enhanced response amplitude across 25 trials in the stimulus set and its effects were more prominent for early trials.

Note that data for all stimulus rates (2–40 Hz) were collected after a single nicotine injection and that stimulus sets at different rates were presented in random order. Moreover, group data (Figure 3B) are based on two stimulus sets for each rate, delivered at different times after the nicotine injection. Thus, the greater effect of nicotine on early trials is not due to stronger effects immediately after the nicotine injection that dissipate over time. Rather, the results suggest a nicotinic effect on auditory processing that strongly enhances initial responses then adapts slowly to a lower level where it remains for the entire stimulus set. The degree of adaptation is estimated by the adaptation ratio (above), whereas the *rate* of adaptation can be estimated by fitting the nicotine data with an exponential decay function (smooth red line in Figure 3B); the decay rates (tau, 2 Hz: 1.6 trials; 5 Hz: 2.5 trials; 10 Hz: 1.0 trials) are similar over a five-fold range of stimulus frequency, suggesting a mechanism independent of frequency.

The effect of nicotine on peak latency across 25 trials is shown in Figure 3C. Control latencies (pre-drug and saline) exhibited trial-to-trial variability that changed little over 25 trials (Figure 3C, left). For 2 and 5 Hz trains, nicotine reduced peak latencies, as expected (cf. Figure 2B), but also reduced trial-to-trial variability so that latencies were more consistent across the stimulus set. We compared variability among conditions by determining the coefficient of variation (CV; Figure 3C, right) and found that nicotine reduced CV (RM-ANOVA; 2 Hz: *p* = 0.0087, *n* = 7; 5 Hz: *p* = 0.0100, *n* = 10). For 10 Hz, peak latencies in control conditions were already short (as described above), exhibited little variability, and were not affected by nicotine (*p* = 0.4443). Note that while slower stimulus rates (2–5 Hz) could generate current sinks with one or two peaks (arrows in Figure 2A, inset trace for 5 Hz), 10 Hz stimuli generated only single, short-latency peaks (Figure 2A, inset trace for 10 Hz stimulus). It appears, therefore, that longer-latency peaks adapt with 10 Hz stimulation and that nicotine does not regulate the latency of the remaining peak (but does regulate its amplitude; Figure 3B).

Overall, this analysis of nicotine’s effects across 25 trials shows that the drug enhances response amplitude for 2–10 Hz trains, as expected (Figure 2A), but responses early in the stimulus set are affected more strongly. Nicotine also reduced response latency, and latency variability, for 2–5 Hz trains so that trial-to-trial consistency was enhanced. Thus, over a stimulus set, nicotine served to enhance response amplitude and consistency, emphasizing response amplitude to initial stimuli in particular. A comparison of within-trial (Figure 2) and across-trial (Figure 3) responses reveals two mechanisms of adaptation: the well-documented, within-trial adaptation is unaffected by nicotine (even as response amplitudes are enhanced), whereas a novel, comparatively slow, across-trial adaptation is prominent only when responses are initially enhanced by the presence of nicotine.

### Nicotine Does Not Affect Train-Evoked Responses in Layer 5/6

**FIGURE 4:**
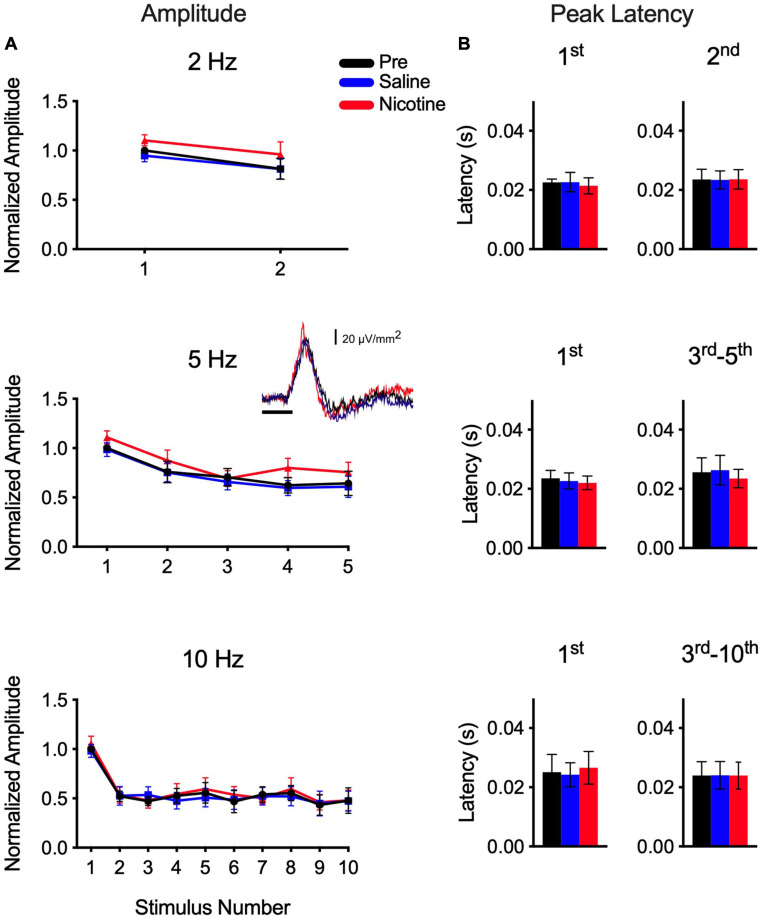
Nicotine had no effect on CF-evoked current sink in layer 5/6. Infragranular current sink recorded simultaneously with current sink in layer 4. **(A)** Systemic nicotine did not affect peak amplitude of responses to stimulus sets (2–10 Hz). Inset shows example “first response” to CF stimulus in layer 5/6. **(B)** Group data for peak latency, separately for first response of train and for adapted responses combined.

Finally, we examined the infragranular CSD profile to determine the effects of systemic nicotine (Figure 4). The CF-evoked layer 5/6 current sink reflects synaptic activity in infragranular neurons (Cruikshank et al., 2002; Zhou et al., 2010), though compared to the layer 4 current sink it is smaller (inset in Figure 4A) and exhibits a shorter-latency peak (Figure 4B). Results for the layer 5/6 current sink were obtained simultaneously with data in layer 4. In response to CF stimulus trains of 2–10 Hz, the layer 5/6 current sink exhibited weaker adaptation (Figure 4A) than observed in layer 4 (Figure 2A). However, unlike in layer 4, systemic nicotine had no effect on either the peak amplitude (Figure 4A) or peak latency (Figure 4B) of the CF-evoked layer 5/6 current sink (RM-ANOVA; *p* ≫ 0.05, *n* = 6–10). It may be worth noting that the peak latency of the layer 5/6 current sink is short (∼20 ms) and similar to that of adapted 10-Hz responses in layer 4 whose latencies also are not affected by nicotine (Figures 2B, 3C). These short-latency responses may reflect a greater contribution of afferent thalamocortical, rather than intracortical, processes.

## Discussion

We examined the effects of systemic nicotine on CSD responses to CF stimulus trains of 2–40 Hz in urethane/xylazine-anesthetized mice. Nicotine generally enhanced the tone-evoked current sink in layer 4 resulting in three novel findings: (i) *within* each one-second trial of CF trains at rates of 2–10 Hz, nicotine enhanced the first response and subsequent, partially adapted responses without affecting the degree of adaptation (adaptation ratio); (ii) *across* 25 trials in each stimulus set, nicotine preferentially enhanced early-trial responses revealing a novel, slower form of adaptation with a time-course of seconds; (iii) across trials, nicotine also enhanced the consistency of response timing for 2–5 Hz trains. Nicotine had no effect on the layer 5/6 current sink in A1, nor on brainstem ABRs. The overall effect of nicotine in layer 4 was to enhance all responses within each trial, to emphasize earlier trials across multiple trials, and to improve the consistency of timing across trials. These effects may improve cortical processing of acoustic streams, such as speech envelopes, that encode information in the 2–10 Hz range.

### Adaptation of CSD Responses Evoked by Acoustic Trains

An advantage of using CSD recordings for this study is that current sinks reflect summed synaptic integration within local circuits rather than simply their output (action potentials) (Muller-Preuss and Mitzdorf, 1984; Metherate et al., 2012). The middle-layer current sink with the earliest onset is considered to be the site of thalamocortical input (designated “layer 4”). However, although the layer 4 current sink is triggered by thalamocortical input, by the time the response reaches peak amplitude at a latency of ∼30–50 ms it is dominated by intracortical activity (Intskirveli et al., 2016). Thus, the layer 4 current sink reflects both monosynaptic thalamic input, especially at short latencies, and progressively greater contributions of intracortical activity at longer latencies. It follows that the preferential adaptation of longer-latency components at moderate stimulus rates (e.g., 10 Hz, Figure 2A) likely reflects the failure of multi-synaptic intracortical activity, whereas adaptation of shorter-latency components at all rates could also reflect reduced thalamic input (Creutzfeldt et al., 1980). The present study demonstrates that current-sink adaptation—which increased with stimulus rate and was complete at ≥ 20 Hz—resembles that described for single units in anesthetized and waking animals (Creutzfeldt et al., 1980; Wang et al., 2008; Yao et al., 2015). Studies in waking animals including humans describe more complex response dynamics in addition to simple adaptation (Todorovic et al., 2011; Phillips et al., 2017), indicating that the present study targets only a subset of mechanisms. Still, the results illustrate the usefulness of CSD recordings for studying nicotinic regulation of adaptation.

In the present study, urethane anesthetic was preferred for its limited depressive effects on nicotinic responses compared to other anesthetics (Hara and Harris, 2002). Still, urethane does reduce sensory cortex responsiveness (Sceniak and Maciver, 2006), though not synaptic activity mediated by glutamate or GABA (Sceniak and Maciver, 2006), and the present studies should be extended to awake animals. However, since nicotine can alter cognition-related electrophysiological responses (Harkrider and Hedrick, 2005), tests in awake animals should control behavioral state as well.

CF train stimuli can be useful for understanding auditory processing since stimuli within an acoustic stream elicit more complex response dynamics than tones in isolation. The present results point to potential consequences of activating nAChRs during an acoustic stream, with the caveat that some effects will likely depend on brain state, especially states such as arousal and attention that are associated with release of endogenous acetylcholine (Celesia and Jasper, 1966; Parikh et al., 2007). That is, dose-dependent effects of nicotine will depend on endogenous, as well as exogenous, activation of nAChRs. Consistent with this notion, studies in human subjects have found that effects of nicotine can vary with baseline measures, i.e., enhancement of performance in subjects with weaker baseline performance, but not in subjects with stronger baselines (Baschnagel and Hawk, 2008; Knott et al., 2014a,b; Behler et al., 2015). Notably, during attention, adaptation is sensitive to “top-down” regulation, being reduced for unexpected stimulus trains and enhanced for expected trains (Todorovic et al., 2011). While it is unclear to what extent top-down regulation is cholinergic, the effects demonstrated in the present study reflect potential mechanisms by which endogenous acetylcholine and/or exogenous nicotine can regulate processing. A better understanding of these mechanisms may be therapeutically useful, e.g., for development of drug treatments for auditory processing deficits (see final section, below).

### Nicotinic Enhancement of Acoustic Train-Evoked Responses

The effects of systemic nicotine on the CF-evoked layer 4 current sink likely involve actions within the auditory thalamocortical pathway and intracortical circuits in A1 (Kawai et al., 2007; Intskirveli and Metherate, 2012; Askew et al., 2017, 2019). Activation of nAChRs within the thalamocortical pathway increases axon excitability to decrease the latency of thalamic-evoked axon spikes and increase the consistency of spike timing (decreased latency CV). Increased synchrony within a population of thalamocortical axons should enhance summation of converging inputs to cortical neurons, thereby enhancing cortical responses. Intracortical actions include recently identified mechanisms by which robust nicotinic excitation of inhibitory interneurons expressing Vasoactive Intestinal Peptide (VIP) produces disinhibition of pyramidal neurons—likely via VIP-interneuron projections to other interneurons—thereby enhancing responsiveness to afferent inputs (Askew et al., 2019). Although nicotine is delivered systemically and nAChRs are found throughout the auditory pathways (Morley and Happe, 2000; Sottile et al., 2017; Noftz et al., 2020), we have not observed effects of systemic nicotine in the auditory brainstem (ABR, present study) or midbrain and thalamus (Askew et al., 2017) that might contribute to enhanced CF-evoked cortical responses [although midbrain and thalamic effects of systemic nicotine do contribute to the narrowing of cortical receptive fields (Askew et al., 2017)].

The present study describes three novel findings: First, systemic nicotine enhanced the peak response (increased amplitude, decreased latency) of partially adapted responses to CF trains at rates of 2–10 Hz (Figure 2). Adaptation *per se* is not affected by nicotine since the adaptation ratio was not altered and nicotine did not prevent complete adaptation at higher rates (20–40 Hz). Thus, nicotine enhances responses to CF trains but does not affect within-trial adaptation (time-course of hundreds of milliseconds).

Second, analysis *across* the 25 trials of each stimulus set revealed a slower adaptation (time-course of seconds), that is evident only in the presence of nicotine to enhance initial responses (Figure 3B). Nicotine does regulate this slower adaptation since it is weak or absent in controls, and the adaptation may depend on nicotinic mechanisms since the adaptation rate is similar across a five-fold range of stimulus frequency. For example, the slow adaptation could involve neuromodulatory mechanisms since nicotinic regulation of tone-evoked responses in A1 requires activation of intracellular MAP kinase (Intskirveli and Metherate, 2012). Previous studies have demonstrated multiple forms of adaptation in A1 with time-courses ranging from hundreds of milliseconds to tens of seconds (Ulanovsky et al., 2004). Similarly, fast and slow forms of adaptation over hundreds of milliseconds and tens of seconds, respectively, can be observed in the *in vitro* auditory cortex with stimulation of afferent inputs (Metherate and Ashe, 1995), suggesting mechanisms that are cortical (or thalamocortical) in origin. Nicotinic regulation of these mechanisms may contribute to top-down regulation of auditory cortex, e.g., during attention-related release of acetylcholine that activates nAChRs.

Third, a striking effect of nicotine in the present study is the enhanced consistency of response timing across trials for 2–5 Hz trains (Figure 3C). That is, nicotine reduced trial-to-trial variability of peak latency even as peak amplitude was enhanced and then adapted. This regulation of peak latency only occurred in responses with longer-latency response components (peak latency ∼30–50 ms), whereas the absence of such responses for 10 Hz trains, likely due to adaptation of intracortical response components, precluded this effect. The nicotinic effect on peak timing is reminiscent of effects on axon spike timing in thalamocortical axons described above (Kawai et al., 2007) and may reflect, in part, increased synchrony of discharge among afferent inputs. Given the importance of timing in auditory processing, it is likely that increased consistency could enhance auditory processing generally.

Finally, in contrast to the effects of nicotine on the layer 4 current sink, we observed no effect on the simultaneously recorded infragranular current sink (layer 5/6; Figure 4) or brainstem responses (ABR; Figure 1B). The infragranular sink exhibits very short latencies (e.g., onset <10 ms, peak ∼20 ms) and likely reflects collateral projections of the main thalamocortical input (Cruikshank et al., 2002; Zhou et al., 2010). Compared to the layer 4 response, the infragranular current sink exhibited weaker adaptation at each stimulus rate tested, and neither its peak amplitude nor peak latency was affected by systemic nicotine. The ABR exhibited no adaptation even at the highest rate tested (40 Hz) and its peak amplitude and latency were not affected by nicotine. Although the ABR recordings were of insufficient resolution to measure individual components, ABR studies in human subjects also found limited effects (reduced Wave I; no effect on Waves III and V) by systemic nicotine (transdermal patch) (Harkrider et al., 2001). However, studies in human subjects did observe nicotinic enhancement (increased amplitude, decreased latency) for longer-latency potentials of presumed thalamocortical and cortical origin (Harkrider and Champlin, 2001) and improved consonant-vowel discrimination measured both behaviorally and electrophysiologically (Harkrider and Hedrick, 2005).

### Implications for Possible Therapeutic Use of Nicotine

In human subjects, cortical activity tracks the envelope of ongoing speech at frequencies ≤10 Hz that correspond to the occurrence of syllables, words and phrases (Vander Ghinst et al., 2019; Fuglsang et al., 2020). Cortical speech tracking is enhanced by attention, and enhanced tracking is associated with better speech comprehension, even as subjects age (Mesgarani and Chang, 2012; Decruy et al., 2019). Indeed, envelop tracking increases more with comprehension in older subjects than in young adults (Decruy et al., 2019), and with hearing loss in older adults (Fuglsang et al., 2020), suggesting that compensatory brain mechanisms enhance speech tracking when the task is more difficult (e.g., with aging and/or hearing loss). Such results raise the possibility that activation of nAChRs by exogenous agonist, including nicotine itself, could help compensate for auditory deficits by increasing the gain and temporal consistency of cortical responses (Metherate et al., 2012), similarly to the findings of the present study. Indeed, recent psychoacoustic studies show performance enhancement with systemic nicotine (polacrilex gum) in normal-hearing young adults, especially in more difficult listening conditions (Pham et al., 2020). Future studies will explore this in human subjects with auditory processing deficits associated with aging or communication disorders.

## Data Availability Statement

The original contributions presented in the study are included in the article/supplementary material, further inquiries can be directed to the corresponding author/s.

## Ethics Statement

The animal study was reviewed and approved by University of California, Irvine, IACUC.

## Author Contributions

II and RM contributed to the conception and design of the study. II collected and analyzed the data. Both authors prepared the manuscript and approved the submitted version.

## Conflict of Interest

The authors declare that the research was conducted in the absence of any commercial or financial relationships that could be construed as a potential conflict of interest.

## References

[B1] Albuquerque E. X. Pereira E. F. Alkondon M. Rogers S. W. (2009). Mammalian nicotinic acetylcholine receptors: from structure to function. *Physiol. Rev.* 89 73–120. 10.1152/physrev.00015.2008 19126755 PMC2713585

[B2] Askew C. Intskirveli I. Metherate R. (2017). Systemic nicotine increases gain and narrows receptive fields in A1 via integrated cortical and subcortical actions. *eNeuro* 4:ENEURO.0192-17.2017. 10.1523/ENEURO.0192-17.2017 PMC5480142 28660244

[B3] Askew C. E. Lopez A. J. Wood M. A. Metherate R. (2019). Nicotine excites VIP interneurons to disinhibit pyramidal neurons in auditory cortex. *Synapse* 73:e22116. 10.1002/syn.22116 PMC6767604 31081950

[B4] Baschnagel J. S. Hawk L. W. Jr. (2008). The effects of nicotine on the attentional modification of the acoustic startle response in nonsmokers. *Psychopharmacology* 198 93–101. 10.1007/s00213-008-1094-y 18338158 PMC2650080

[B5] Behler O. Breckel T. P. Thiel C. M. (2015). Nicotine reduces distraction under low perceptual load. *Psychopharmacology* 232 1269–1277. 10.1007/s00213-014-3761-5 25304866

[B6] Celesia G. G. Jasper H. H. (1966). Acetylcholine released from cerebral cortex in relation to state of activation. *Neurology* 16 1053–1070. 10.1212/wnl.16.11.1053 5950916

[B7] Creutzfeldt O. Hellweg F. C. Schreiner C. (1980). Thalamocortical transformation of responses to complex auditory stimuli. *Exp. Brain Res.* 39 87–104. 6247179 10.1007/BF00237072

[B8] Cruikshank S. J. Rose H. J. Metherate R. (2002). Auditory thalamocortical synaptic transmission in vitro. *J. Neurophysiol.* 87 361–384. 10.1152/jn.00549.2001 11784756

[B9] Dani J. A. Bertrand D. (2007). Nicotinic acetylcholine receptors and nicotinic cholinergic mechanisms of the central nervous system. *Annu. Rev. Pharmacol. Toxicol.* 47 699–729. 10.1146/annurev.pharmtox.47.120505.105214 17009926

[B10] Decruy L. Vanthornhout J. Francart T. (2019). Evidence for enhanced neural tracking of the speech envelope underlying age-related speech-in-noise difficulties. *J. Neurophysiol.* 122 601–615. 10.1152/jn.00687.2018 31141449 PMC6734401

[B11] Fuglsang S. A. Marcher-Rorsted J. Dau T. Hjortkjaer J. (2020). Effects of sensorineural hearing loss on cortical synchronization to competing speech during selective attention. *J. Neurosci.* 40 2562–2572. 10.1523/jneurosci.1936-19.2020 32094201 PMC7083526

[B12] Hara K. Harris R. A. (2002). The anesthetic mechanism of urethane: the effects on neurotransmitter-gated ion channels. *Anesth. Analg.* 94 313–318. 10.1097/00000539-200202000-00015 11812690

[B13] Harkrider A. W. Champlin C. A. (2001). Acute effect of nicotine on non-smokers: II. MLRs and 40-Hz responses. *Hear. Res.* 160 89–98. 10.1016/s0378-5955(01)00346-x 11591494

[B14] Harkrider A. W. Champlin C. A. (2001). Acute effect of nicotine on non-smokers: III. LLRs and EEGs. *Hear. Res.* 160 99–110. 10.1016/s0378-5955(01)00347-1 11591495

[B15] Harkrider A. W. Champlin C. A. McFadden D. (2001). Acute effect of nicotine on non-smokers: I. OAEs and ABRs. *Hear. Res.* 160 73–88. 10.1016/s0378-5955(01)00345-8 11591493

[B16] Harkrider A. W. Hedrick M. S. (2005). Acute effect of nicotine on auditory gating in smokers and non-smokers. *Hear. Res.* 202 114–128. 10.1016/j.heares.2004.11.009 15811704

[B17] Intskirveli I. Joshi A. Vizcarra-Chacon B. J. Metherate R. (2016). Spectral breadth and laminar distribution of thalamocortical inputs to A1. *J. Neurophysiol.* 115 2083–2094. 10.1152/jn.00887.2015 26888102 PMC4869500

[B18] Intskirveli I. Metherate R. (2012). Nicotinic neuromodulation in auditory cortex requires MAPK activation in thalamocortical and intracortical circuits. *J. Neurophysiol.* 107 2782–2793. 10.1152/jn.01129.2011 22357798 PMC3362282

[B19] Kawai H. Lazar R. Metherate R. (2007). Nicotinic control of axon excitability regulates thalamocortical transmission. *Nat. Neurosci.* 10 1168–1175. 10.1038/nn1956 17704774

[B20] Knott V. Choueiry J. Dort H. Smith D. Impey D. de la Salle S. (2014a). Baseline-dependent modulating effects of nicotine on voluntary and involuntary attention measured with brain event-related P3 potentials. *Pharmacol. Biochem. Behav.* 122 107–117. 10.1016/j.pbb.2014.03.020 24690514

[B21] Knott V. Impey D. Philippe T. Smith D. Choueiry J. de la Salle S. (2014b). Modulation of auditory deviance detection by acute nicotine is baseline and deviant dependent in healthy nonsmokers: a mismatch negativity study. *Hum. Psychopharmacol.* 29 446–458. 10.1002/hup.2418 25196041

[B22] Mesgarani N. Chang E. F. (2012). Selective cortical representation of attended speaker in multi-talker speech perception. *Nature* 485 233–236. 10.1038/nature11020 22522927 PMC3870007

[B23] Metherate R. Ashe J. H. (1995). GABAergic suppression prevents the appearance and subsequent fatigue of an NMDA receptor-mediated potential in neocortex. *Brain Res.* 699 221–230. 10.1016/0006-8993(95)00909-a 8616625

[B24] Metherate R. Intskirveli I. Kawai H. D. (2012). Nicotinic filtering of sensory processing in auditory cortex. *Front. Behav. Neurosci.* 6:44. 10.3389/fnbeh.2012.00044 PMC3400128 22833720

[B25] Morley B. J. Happe H. K. (2000). Cholinergic receptors: dual roles in transduction and plasticity. *Hear. Res.* 147 104–112. 10.1016/s0378-5955(00)00124-6 10962177

[B26] Muller-Preuss P. Mitzdorf U. (1984). Functional anatomy of the inferior colliculus and the auditory cortex: current source density analyses of click-evoked potentials. *Hear. Res.* 16 133–142. 10.1016/0378-5955(84)90003-0 6526745

[B27] Noftz W. A. Beebe N. L. Mellott J. G. Schofield B. R. (2020). Cholinergic projections from the pedunculopontine tegmental nucleus contact excitatory and inhibitory neurons in the inferior colliculus. *Front. Neural Circuits* 14:43. 10.3389/fncir.2020.00043 32765226 PMC7378781

[B28] Parikh V. Kozak R. Martinez V. Sarter M. (2007). Prefrontal acetylcholine release controls cue detection on multiple timescales. *Neuron* 56 141–154. 10.1016/j.neuron.2007.08.025 17920021 PMC2084212

[B29] Pham C. Q. Kapolowicz M. R. Metherate R. Zeng F. G. (2020). Nicotine enhances auditory processing in healthy and normal-hearing young adult nonsmokers. *Psychopharmacology* 237 833–840. 10.1007/s00213-019-05421-x 31832719 PMC7039769

[B30] Phillips E. A. K. Schreiner C. E. Hasenstaub A. R. (2017). Diverse effects of stimulus history in waking mouse auditory cortex. *J. Neurophysiol.* 118 1376–1393. 10.1152/jn.00094.2017 28566458 PMC5558031

[B31] Poorthuis R. B. Bloem B. Verhoog M. B. Mansvelder H. D. (2013). Layer-specific interference with cholinergic signaling in the prefrontal cortex by smoking concentrations of nicotine. *J. Neurosci.* 33 4843–4853. 10.1523/jneurosci.5012-12.2013 23486955 PMC6618989

[B32] Sceniak M. P. Maciver M. B. (2006). Cellular actions of urethane on rat visual cortical neurons in vitro. *J. Neurophysiol.* 95 3865–3874. 10.1152/jn.01196.2005 16510775

[B33] Sottile S. Y. Ling L. Cox B. C. Caspary D. M. (2017). Impact of ageing on postsynaptic neuronal nicotinic neurotransmission in auditory thalamus. *J. Physiol.* 595 5375–5385. 10.1113/jp274467 28585699 PMC5538226

[B34] Stiebler I. Neulist R. Fichtel I. Ehret G. (1997). The auditory cortex of the house mouse: left-right differences, tonotopic organization and quantitative analysis of frequency representation. *J. Comp. Physiol. A* 181 559–571. 10.1007/s003590050140 9449817

[B35] Todorovic A. van Ede F. Maris E. de Lange F. P. (2011). Prior expectation mediates neural adaptation to repeated sounds in the auditory cortex: an MEG study. *J. Neurosci.* 31 9118–9123. 10.1523/jneurosci.1425-11.2011 21697363 PMC6623501

[B36] Ulanovsky N. Las L. Farkas D. Nelken I. (2004). Multiple time scales of adaptation in auditory cortex neurons. *J. Neurosci.* 24 10440–10453. 10.1523/jneurosci.1905-04.2004 15548659 PMC6730303

[B37] Vander Ghinst M. Bourguignon M. Niesen M. Wens V. Hassid S. Choufani G. (2019). Cortical tracking of speech-in-noise develops from childhood to adulthood. *J. Neurosci.* 39 2938–2950. 10.1523/jneurosci.1732-18.2019 30745419 PMC6462442

[B38] Wang X. Lu T. Bendor D. Bartlett E. (2008). Neural coding of temporal information in auditory thalamus and cortex. *Neuroscience* 154 294–303. 10.1016/j.neuroscience.2008.03.065 18555164 PMC2751884

[B39] Yao J. D. Bremen P. Middlebrooks J. C. (2015). Emergence of spatial stream segregation in the ascending auditory pathway. *J. Neurosci.* 35 16199–16212. 10.1523/jneurosci.3116-15.2015 26658870 PMC4682785

[B40] Zhou Y. Liu B. H. Wu G. K. Kim Y. J. Xiao Z. Tao H. W. (2010). Preceding inhibition silences layer 6 neurons in auditory cortex. *Neuron* 65 706–717. 10.1016/j.neuron.2010.02.021 20223205 PMC2904750

